# Long-Term Exercise Alters the Profiles of Circulating Micro-RNAs in the Plasma of Young Women

**DOI:** 10.3389/fphys.2020.00372

**Published:** 2020-05-08

**Authors:** Fan Li, Muwei Bai, Jianfang Xu, Ling Zhu, Chengyi Liu, Rui Duan

**Affiliations:** ^1^Laboratory of Regenerative Medicine in Sports Science, School of Physical Education and Sports Science, South China Normal University, Guangzhou, China; ^2^Laboratory of Laser Sports Medicine, School of Physical Education and Sports Science, South China Normal University, Guangzhou, China; ^3^Department of Physical Education, Guangdong Pharmaceutical University, Guangzhou, China; ^4^China Institute of Sport Science, Beijing, China

**Keywords:** long-term exercise, circulating miRNAs, risk of cancer, human plasma, women

## Abstract

**Objective:** The objective of this paper was to study the effects of long-term exercise on circulating microRNAs (miRNAs) in human plasma.

**Methods:** Whole blood was collected from 10 female elite athletes with at least 5 years of training experience in a Synchronized Swimming Group (S group) and 15 female college students without regular exercise training (C group). Plasma miRNAs were then isolated, sequenced, and semi-quantified by the second-generation sequencing technology, and the results were analyzed by bioinformatics methods.

**Results:** We found 380 differentially expressed miRNAs in the S group compared with the C group, among which 238 miRNAs were upregulated and 142 were downregulated. The top five abundant miRNAs in the 380 miRNAs of the S group are hsa-miR-451a, hsa-miR-486, hsa-miR-21-5p, hsa-miR-423-5p, and hsa-let-7b-5p. Muscle-specific/enriched miRNAs were not significantly different, except for miR-206 and miR-486. According to the Kyoto Encyclopedia of Genes and Genomes (KEGG) pathway analysis, a large proportion of the differentially expressed miRNAs are targeted in cancer-related pathways, including proteoglycans in cancer and miRNAs in cancer and basal cell carcinoma. As the levels of circulating miRNAs (ci-miRNAs) are commonly known to be significantly deregulated in cancer patients, we further compared the levels of some well-studied miRNAs in different types of cancer patients with those in the S group and found that long-term exercise regulates the level of ci-miRNAs in an opposite direction to those in cancer patients.

**Conclusion:** Long-term exercise significantly alters the profiles of plasma miRNAs in healthy young women. It may reduce the risk of certain types of cancers by regulating plasma miRNA levels.

**What are the findings?**

• Long-term exercise significantly alters the plasma miRNA profile of healthy young women.
• Long-term exercise may reduce the risk of lung, breast, pancreas, esophageal, thyroid neoplasms, melanoma and cholangiocarcinoma by regulating circulating miRNAs.
• Athletes may have a higher risk of heart failure and coronary artery disease.

## Introduction

MicroRNAs (miRNAs) are small non-coding RNAs with approximately 22 nucleotides in length. Their function is to degrade the targeted messenger RNAs (mRNAs) or inhibit their translation by binding to the complementary region of the mRNA molecules, which in turn participate in various biological or pathological processes (Bartel, 2004). They are found to be abundant and stable in biofluids, including blood serum/plasma (Chim et al., 2008), cerebrospinal fluid (CSF) (Baraniskin et al., 2012; Sorensen et al., 2014; Akers et al., 2015), milk (Modepalli et al., 2014), saliva (Bahn et al., 2015), and urine (Cheng et al., 2014). These miRNAs in the biofluids are called circulating miRNAs (ci-miRNAs) and may come from dead cells or as by-products of routine microvesicle secretion or specifically secreted by cells (Turchinovich et al., 2012). The levels of ci-miRNAs will change under different physio- or pathological conditions, such as pregnancy (Sanders et al., 2015), myocardial infarction (Boon and Dimmeler, 2015), muscle injury (Desai et al., 2014), liver damage (Leelahavanichkul et al., 2015), diabetes (Ortega et al., 2014), and cancer (Volinia et al., 2010).

A few studies have shown that plasma ci-miRNAs can also be affected by a single bout of acute exercise (Baggish et al., 2011; Aoi et al., 2013; Nielsen et al., 2014; Cui et al., 2016; Shah et al., 2017; Barber et al., 2019) or sustained training from 28 to 140 days (Baggish et al., 2011; Aoi et al., 2013; Nielsen et al., 2014; Li et al., 2018; Barber et al., 2019). Most of them focused on some specific ci-miRNAs, such as ci-miRNAs involved in inflammation, skeletal and cardiac muscle contractility, and hypoxia/ischemia adaptation (Baggish et al., 2011), muscle-enriched miRNAs (Aoi et al., 2013), miRNAs related to cardiovascular disease (Barber et al., 2019), and those involved in angiogenesis and inflammation and are enriched in muscle and/or cardiac tissues (Li et al., 2018). Only one study (Nielsen et al., 2014) quantified the expression levels of 188 known ci-miRNAs in the plasma of 32 healthy, trained men before and after 12 weeks of endurance exercise and found nine miRNAs expressed differently. By far, the response of whole ci-miRNAs in plasma to long-term exercise (>5 years), especially among long-term trained professional athletes, remains unknown. In this study, we chose athletes of a Synchronized Swimming Group as our subjects since the movement difference between players is small and the team members usually have years of regular exercise experience. We compared the total plasma miRNAs of 10 players from a Synchronized Swimming Group and 15 female college students without regular exercise experience, using second-generation sequencing technology, to study the impact of long-term exercise on the plasma miRNA content.

## Materials and Methods

### Subjects

The participants were 10 Synchronized Swimming Group members and 15 female college students of Guangdong Pharmaceutical University. All participants were informed of the methods, procedures, and risks, and then they signed an informed consent document, which was approved by the Ethics Committee of South China Normal University School of Sports Science (approval number 2017102001).

### MiRNA Isolation From Plasma

Blood samples were collected around 7:00 a.m., and participants did not have breakfast or vigorous exercise for 24 h. Whole blood (10 ml) from subjects was collected *via* a direct venous puncture into tubes with ethylenediaminetetraacetic acid (EDTA) as an anticoagulant. Blood was put on ice for half an hour and then separated by 2,000 rpm for 10 min at 4°C. Then, the plasma was transferred to an RNase-free tube and stored at −80°C. The RNA samples were extracted with a TRIzol reagent (Life Technologies, cat. no. 15596018). The purity and concentration of the RNA samples were assessed with Nanodrop and Qubit 2.0. RNA integrity was assessed using the RNA Nano 6000 Assay Kit of the Agilent Bioanalyzer 2100 system (Agilent Technologies, Santa Clara, CA, United States) to ensure the use of qualified samples for sequencing.

### MiRNA Sequencing

RNA samples undergo a series of strict quality control. Qualified samples are used for library construction. Library construction is strictly in accordance with NEBNext Ultra Small RNA Sample Library Prep Kit for Illumina, qualified libraries for high-throughput sequencing. The sequencing platform was Illumina HiSeq X Ten, and read length was single-end (SE) 50 nt.

### Data Analysis

#### Quality Control

Raw data (raw reads) of fastq format were firstly processed through in-house perl scripts. In this step, clean data (clean reads) were obtained by removing reads containing adapter or ploy-N and low-quality reads from raw data. The reads were trimmed and cleaned by removing the sequences smaller than 15 nt or longer than 35 nt. At the same time, Q20, Q30, GC content, and the sequence duplication level of the clean data were calculated. All the downstream analyses were based on clean data with high quality.

#### Comparative Analysis

Using Bowtie software, analysis of the clean reads was done, respectively, with the Silva database, GtRNAdb database, Rfam database, and Repbase database for sequence alignment, filter ribosomal RNA (rRNA), transfer RNA (tRNA), small nuclear RNA (snRNA), small nucleolar RNA (snoRNA), and other ncRNAs and repeats. The remaining reads were used to detect known miRNAs and novel miRNAs predicted by comparing with Genome and known miRNAs from miRBase. Randfold software was used for novel miRNA secondary structure prediction.

#### Differential Expression Analysis

The expression of miRNAs in each sample was counted and normalized by the Trusted Platform Module (TPM) algorithm (Fahlgren et al., 2007). The normalization formula for TPM is as follows: TPM = Actual miRNA read count/Total miRNA read count × 1,000,000. Differential expression analysis was performed using the DESeq R package (1.10.1). The resulting *P* values were adjusted using the Benjamini and Hochberg’s approach for controlling the false discovery rate (FDR). MiRNAs with |log_2_(FC, fold change)| ≥ 1 and FDR < 0.05 found by DESeq were assigned as differentially expressed miRNAs.

#### Target Gene Functional Annotation

Target gene prediction was done using miRanda (Doron Betel et al., 2008) and RNAhybrid (Rehmsmeier et al., 2004). Gene function was annotated based on the following databases: Nr (NCBI non-redundant protein sequences), Pfam (Protein family), KOG/COG (Clusters of Orthologous Groups of proteins), Swiss-Prot (a manually annotated and reviewed protein sequence database), KEGG (KEGG Ortholog database), and GO (Gene Ontology).

#### Enrichment Analysis

A GO term enrichment analysis^1^ was used to identify the biological processes, molecular functions, and cellular components associated with the target genes of differential expression miRNAs. KEGG pathway enrichment analysis^2^ was used to identify the miRNA targets associated with signaling pathways in the S vs. the C group.

#### MiRNAs as Biomarkers of Certain Diseases (Study Selection Criteria)

We screened ci-miRNA biomarkers related to certain types of cancers through the Human MicroRNA Disease Database (HMDD^3^). Studies were included if they (i) were original research and (ii) evaluated the ci-miRNA levels in a specific disease. Studies were excluded if they were published in a language other than English and are review articles, book chapters, conference abstracts, editorials/commentaries/expert opinion, theses, or dissertations.

## Results

### The Expression Patterns of Ci-MiRNAs Were Highly Similar Within the Originally Divided Participants

**FIGURE 1:**
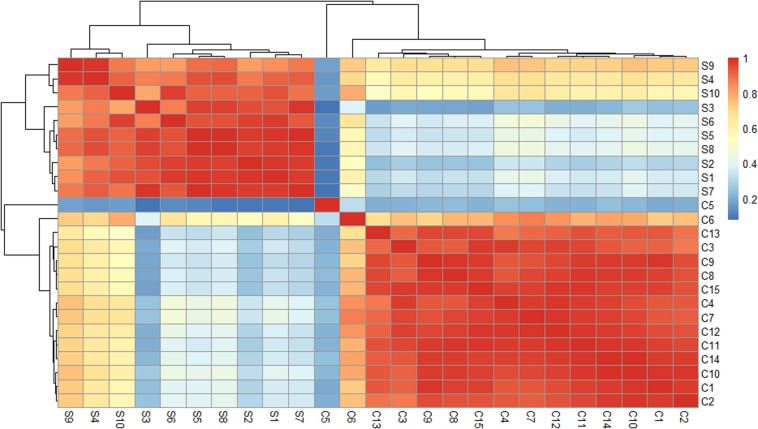
Heat maps of the Pearson’s correlation coefficients for different samples. When the linear relationship between the two variables is enhanced, the correlation coefficient tends to be 1 or –1. A positive correlation tends to be 1, while a negative correlation tends to be –1.

The participants included 10 Synchronized Swimming Group members (S group) and 15 female college students from Guangdong Pharmaceutical University in China (C group). The average age of these 10 players was 19.1 years compared with 19.4 years of the control, and there was no significant age difference between these two groups (19.1 ± 2.08 vs. 19.4 ± 0.83 years, *P* = 0.67). The subjects were all healthy and without known diseases. C group members had no exercise habit and did not enroll in any long-term sports or dancing in the last 5 years. For details, see Supplementary Table 1. Pearson’s correlation coefficient was used to test the similarity of these samples at a global level (Figure 1). The heat map of the correlations between each sample was generated and showed distinct expression pattern between the two groups. The clustering of samples showed that the same group of samples is very similar to the groups of samples originally divided.

### Differentially Expressed MiRNAs Between the S and C Groups

**FIGURE 2:**
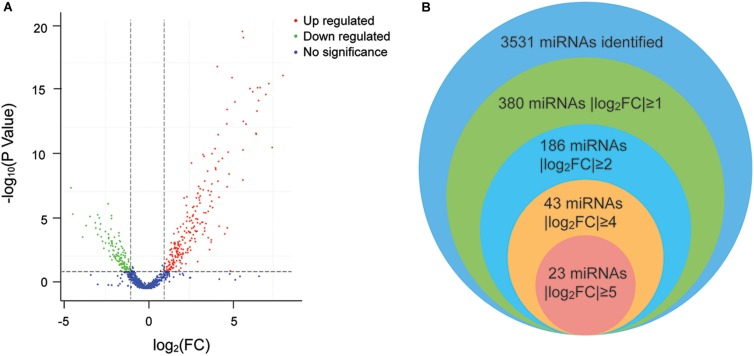
**(A)** Volcano plot of the differentially expressed miRNAs. The plot shows the log_2_ fold change on the *X*-axis vs. the adjusted *P* values (on the log10 scale) on the *Y*-axis. *Red dots* indicate upregulated miRNAs, *green dots* are downregulated miRNAs, and *blue dots* are miRNAs with no significant difference. **(B)** Venn diagram of the differentially expressed miRNAs. Each *circle* represents a comparison group. The *overlapping part* is the common miRNAs among the comparison groups.

Twenty-five specimens were successfully sequenced by using Illumina Genome Analyzer II. The number of raw reads obtained per specimen ranged from 15,872,436 to 32,103,564 (average = 23,148,465). From the raw reads, an average of 22,508,866 clean reads (ranging from 15,569,728 to 31,145,335) were filtered and mapped to human genome. After analyzing and normalizing all the mapped reads in both groups, we identified the existence of 3,531 miRNAs with 1,733 known miRNAs and 1,798 novel miRNAs. Then, we applied a stringent filtering criterion to differentiate S and C (FDR < 0.05, | log_2_(FC)| ≥ 1) and identified 380 miRNAs that were differentially expressed (for details, see Supplementary Table 2). Specifically, 238 miRNAs were upregulated and 142 were downregulated (Figure 2A). In addition, 23 miRNAs have an extremely significant difference between the two groups (| log_2_(FC)| ≥ 5) (Figure 2B). Notably, all 23 miRNAs were upregulated in the S group, and the highest differentially expressed miRNA was novel-miR-1150 (| log_2_(FC)| = 8.09). Among the 380 differentially expressed miRNAs, the five most abundant in the S group were miR-451a, miR-486, miR-423-5p, let-7b-5p, and miR-21-5p, with | log_2_(FC)| = 4.77, 4.76, 2.13, 2.65, and 1.53 and TPM = 224,122, 119,250, 85,314, 43,867, and 26,667, respectively. On the other hand, the five most abundant miRNAs in the C group were miR-21-5p, let-7f-5p, miR-148a-3p, miR-423-5p, and miR-146a-5p. Taken together, there are consistent and significant differences in the plasma miRNA profiles between the athlete group and the normal college student group.

### Target Gene Prediction of Differentially Expressed MiRNAs

To explore the functions of chronic exercise induced differentially expressed miRNAs, the target genes of these miRNAs were predicted by combining miRanda and RNAhybrid to reduce the probability of false positives. From this analysis, 20,449 target genes corresponding to 3,531 miRNAs were identified. For details, see Supplementary Table 3.

### Functional Annotation Analysis

Gene ontology enrichment analysis of the 380 differentially expressed genes (DEGs) was implemented by the GOseq R packages based on Wallenius’ non-central hyper-geometric distribution. In the S group, 1,162 GO categories of biological processes, 142 of cellular component, and 203 of molecular function were found to be significantly affected (Kolmogorov–Smirnov, KS < 0.05). The 10 most significantly enriched GO categories of biological processes, cellular component, and molecular function are listed in Table 1.

**TABLE 1:** Top 10 most significantly enriched GO categories of the differentially expressed miRNAs.

GO ID	Term	Annotated	Significant	Expected	KS
**Biological processes**					
GO:0007411	Axon guidance	397	377	347.57	1.9E-12
GO:0071586	CAAX-box protein processing	26	26	22.76	7.6E-10
GO:1900246	Positive regulation of RIG-I signaling pathway	27	27	23.64	3.7E-09
GO:0045944	Positive regulation of transcription by RNA polymerase II	849	793	743.3	6.2E-09
GO:1900245	Positive regulation of MDA-5 signaling pathway	26	26	22.76	9E-09
GO:0050691	Regulation of defense response to virus by host	55	54	48.15	1.1E-08
GO:0031064	Negative regulation of histone deacetylation	25	25	21.89	2.2E-08
GO:0090315	Negative regulation of protein targeting to membrane	27	26	23.64	3.8E-08
GO:0090084	Negative regulation of inclusion body assembly	12	11	10.51	6.4E-07
GO:0001525	Angiogenesis	406	386	355.45	1.00E-06
**Molecular functions**					
GO:0030054	Cell junction	879	831	767.72	2.30E-08
GO:0005737	Cytoplasm	10,110	9,044	8,830.1	5.80E-08
GO:0005667	Transcription factor complex	353	330	308.31	3.60E-07
GO:0005634	Nucleus	6,542	5,808	5,713.8	4.00E-06
GO:0045202	Synapse	559	520	488.23	1.70E-05
GO:0005583	Fibrillar collagen trimer	26	26	22.71	3.10E-05
GO:0005604	Basement membrane	99	95	86.47	4.60E-05
GO:0005874	Microtubule	380	355	331.89	6.30E-05
GO:0072357	PTW/PP1 phosphatase complex	13	13	11.35	8.50E-05
GO:0045211	Postsynaptic membrane	213	199	186.03	0.00017
**Cellular components**					
GO:0043565	Sequence-specific DNA binding	746	711	651.87	3.80E-11
GO:0003700	DNA-binding transcription factor activity	1,119	1,034	977.8	7.80E-09
GO:0008134	Transcription factor binding	536	494	468.37	7.10E-08
GO:0046872	Metal ion binding	4,280	3,837	3,739.9	8.60E-08
GO:0044212	Transcription regulatory region DNA binding	406	396	354.77	1.60E-06
GO:0003779	Actin binding	381	368	332.92	9.00E-06
GO:0005524	ATP binding	1,605	1,458	1,402.5	1.70E-05
GO:0005515	Protein binding	8,322	7456	7,271.9	1.90E-05
GO:0005543	Phospholipid binding	533	500	465.75	2.00E-05
GO:0005488	Binding	12,839	11,463	11,219	2.10E-05

**GO.ID*, ID of the GO term; *Term*, GO function description; *Annotated*, the number of genes annotated with this function; *Significant*, the number of differentially expressed miRNA target genes annotated to this function; *Expected*, the expected number of differentially expressed miRNA target genes annotated to this function; *KS* statistical significance of the enrichment term (the smaller the KS value, the more significant the enrichment).*

### Pathway Annotation Analysis

**FIGURE 3:**
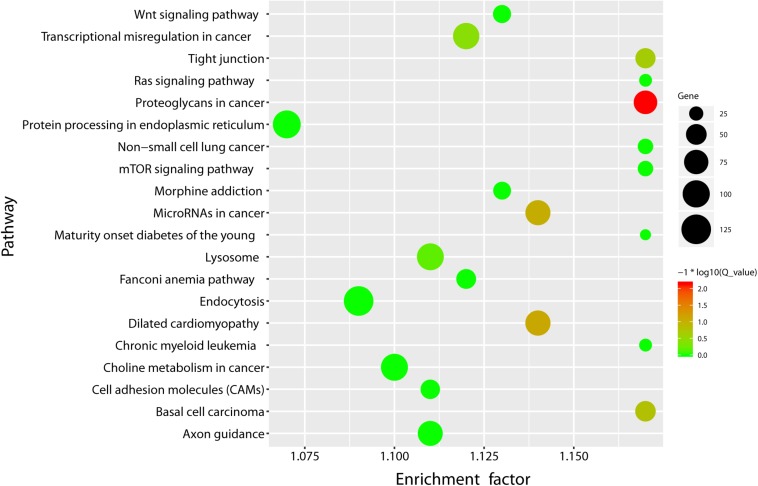
KEGG pathway enrichment bubble plot of the differentially expressed miRNAs. *X*-axis label represents rich factor (rich factor = amount of differentially expressed genes enriched in the pathway/amount of all genes in the background gene set) and *Y*-axis label represents pathway. The *size* and *color of the bubble* represent the amount of differentially expressed genes enriched in the pathway and the enrichment significance (*Q*_ value is the *P* value corrected after multiple hypothesis testing), respectively.

Pathway annotation analysis of the differentially expressed miRNA target genes was done using the KEGG database see text footnote 2). The results are shown in Figure 3.

### Circulating MiRNAs as Biomarkers of Different Diseases

We screened ci-miRNA biomarkers related to certain types of diseases through the HMDD. The results are shown in Table 2.

**TABLE 2:** Ci-miRNA biomarkers related to certain diseases (HMDD database).

	miR-21	miR-146a	miR-126	miR-423	Risk
Lung neoplasms	UP (Liu et al., 2012; Yang et al., 2015; Munagala et al., 2016)				↓
Breast neoplasms	UP (Yan et al., 2008; Schwarzenbach et al., 2012; Kumar et al., 2013; Si et al., 2013; Anfossi et al., 2014; Igglezou et al., 2014; Marino et al., 2014; Matamala et al., 2015; Toraih et al., 2015; Markou et al., 2016; Motawi et al., 2016; Swellam et al., 2018)	UP (Kumar et al., 2013; Markou et al., 2016)			↓
Pancreatic neoplasms	UP (Ali et al., 2010; Abue et al., 2015; Skrha et al., 2015; Goto et al., 2018)				↓
Melanoma		UP (Leidinger et al., 2010; Russo et al., 2016)			↓
Cholangiocarcinoma	UP (Silakit et al., 2017; Liu et al., 2018)				↓
Esophageal neoplasms	UP (Cai et al., 2012; Kurashige et al., 2012; Tanaka et al., 2013)				↓
Colorectal carcinoma	UP (Kanaan et al., 2012; Basati et al., 2014; Wang et al., 2014; Yamada et al., 2015; Zhu et al., 2017)				↓
Coronary artery disease			DOWN (Fichtlscherer et al., 2010; Wang et al., 2017)		↑
Heart failure				UP (Tijsen et al., 2010; Goren et al., 2012; Dickinson et al., 2013)	↑
S group vs. C group	DOWN	DOWN	DOWN	UP	

*UP and DOWN represent the miRNA being increased or decreased, respectively, in the indicated disease compared with the control. “↓,” “↑,” or “—” indicate the risk that exercise may reduce, increase, or unknown, respectively.*

## Discussion

### Differentially Expressed MiRNAs in Athletes Are Likely Due to Long-Term Exercise

As shown in Figure 1, the expression profiles of the ci-miRNAs in athletes were significantly different from those of college students who had not received any regular sports training. Notably, all the participants from both groups in this study come from different parts of China, eating different food. In addition, there are no immediate training seasons prior to the blood withdrawal in the athlete group. In addition to synchronized swimming, the athletes also underwent strength training and aerobic and anaerobic training. Thus, the differentially expressed miRNAs in the athlete group may be a result of combined exercise training. Altogether, the ci-miRNAs’ profile changes in the athlete group are highly likely due to long-term exercise.

### Ci-MiRNA Profile Changes in Athletes Are Correlated With Muscle-Enriched MiRNAs

A set of miRNAs, called myo-miRs, have been identified in skeletal muscle and/or the myocardium, including miR-1, miR-133a, miR-133b, miR-206, miR-208a, miR-208b, and miR-499 (McCarthy and Esser, 2007; Callis et al., 2008; van Rooij et al., 2008; Small et al., 2010). Their expression is at least 20-fold higher than the mean expression value of the other tissues (Lee et al., 2008). Among them, miR-206 is skeletal muscle-specific (McCarthy, 2008) and miR-208a is cardiac muscle-specific (Sempere et al., 2004). MiR-486 is also a skeletal muscle and cardiac-enriched miRNA, which was included in myo-miRs in later studies (Horak et al., 2016). In our study, miR-1 was not detected in all samples. the TPMs of the remaining myo-miRs are shown in Table 3.

**TABLE 3:** Expression level changes of myo-miRs in the athlete group.

miRNAs	TPM (S group)	TPM (C group)	Regulated
miR-133a-3p	18.7	28.5	No
miR-133b	0.43	0.63	No
miR-206	11.7	5.26	Up
miR-208	0.02	0	No
miR-208b	0.15	0.2	No
miR-486-3p	688.45	62.86	Up
miR-486	119,167.2	7,499.67	Up
miR-499	17.81	8.22	No

To test whether the level changes of the other differently expressed miRNAs were associated with muscle-specific/enriched miRNAs, we first calculated a Pearson’s correlation coefficient between miR-486 and the other differently expressed miRNAs. As a myo-miR, miR-486 mainly functions as a regulator of myoblast proliferation and migration (Alexander et al., 2011) and skeletal muscle size (Hitachi et al., 2014). The results showed that a large portion of the differently expressed miRNAs (>30%, R^2^ > 0.6) were highly correlated with miR-486-5p and miR-486-3p (for details, see Supplementary Table 4), which provide another evidence that the miRNA profile changes in the athlete group were likely to be induced by exercise.

Muscle injures are common among athletes. In the plasma of healthy young men, the expression level of miR-206 was significantly higher after high-intensity intermittent exercise (HIIE) or vigorous-intensity continuous exercise (VICE) (Cui et al., 2016). miR-206 is also increased in the serum of mdx and CXMDJ mice (Mizuno et al., 2011) or in response to cardiotoxin (CTX)-induced injury, but markedly decreased from day 3 to day 5 after CTX injury (Matsuzaka et al., 2014). This suggests that high levels of miR-206 in plasma may be associated with skeletal muscle injury. In addition, miR-133 has also been demonstrated to be associated with skeletal muscle injury (Laterza et al., 2009). In the present study, miR-206 was slightly increased in the S group (|log_2_FC| = 1.99, TPM = 0–42.44), and miR-133 did not show significant difference between the two groups. To examine whether the level changes of miRNAs in the athlete group were also associated with muscle injury, we also calculated Pearson’s correlation coefficient between miR-206 and the other differently expressed miRNAs and found that there were less than 3% of the differentially expressed miRNAs correlated with muscle injury (<3%, R^2^ > 0.6; for details, see Supplementary Table 4).

### Long-Term Exercise May Reduce the Risk of Cancers by Regulating Ci-MiRNAs

Physical activity has been shown to be associated with lower cancer risks. From 30 to 60 min/day of moderate- to vigorous-intensity physical activity can decrease the risk of breast cancer, and physically active individuals have a lower risk of lung cancer (Lee, 2003). The overall cancer incidence was lower in athletes than in the general population (Robsahm et al., 2010; Sormunen et al., 2014). And former college athletes had a significantly lower risk of breast cancer than do the non-athletes (Wyshak and Frisch, 2000). However, the underlying mechanism regarding exercise reducing cancer risks remains to be discovered. Lately, a number of studies have indicated that some of the ci-miRNAs (see Table 2) can be used as biomarkers for cancer or other diseases (Komatsu et al., 2014; Zhang et al., 2016; Hara et al., 2017; Khalighfard et al., 2018). Strikingly, long-term exercise regulates the levels of these ci-miRNAs in an opposite direction to those in cancer patients, which suggests that long-term exercise may reduce the risk of cancers through regulating ci-miRNAs.

Interestingly, some of the elevated miRNAs, such as miR-126 and miR-423, in heart failure and coronary artery disease (CAD) were also upregulated in the athlete group. Coronary artery calcification (CAC) is a strong predictor of incident CAD (Detrano et al., 2008). In a prospective study, 27% higher odds of CAC have been seen among participants who exceeded physical activity guidelines vs. those below physical activity guidelines (Laddu et al., 2017). CAD is also a leading cause of sudden cardiac death in athletes over 35 years of age (Morrison et al., 2018). But the incidence rates of sports-related sudden cardiac deaths in noncompetitive and competitive athletes are not different (Risgaard et al., 2014). Does high-intensity or volume exercise increase the risk of CAD? Is miR-126 not a solid biomarker of CAD? Further research is needed to address these questions.

## Conslusion

Long-term exercise significantly alters the plasma miRNA profiles in healthy young women, which may reduce the risk of lung, breast, pancreas, melanoma, cholangiocarcinoma, esophageal neoplasms, and colorectal carcinoma. Our current study described an overall effect of long-term exercise on circulating miRNA profiles in the plasma of young women. Further studies are needed to determine whether different types of exercises have different effects on ci-miRNAs and whether there are gender and age differences.

## Data Availability Statement

The sequencing data has been deposited in the BioProject database (accession: PRJNA613853, https://www.ncbi.nlm.nih.gov/sra?linkname=bioproject_sra_all&from_uid=613853).

## Ethics Statement

The studies involving human participants were reviewed and approved by the Ethics Committee of South China Normal University School of Sports Science (Approval Number 2017102001). Written informed consent to participate in this study was provided by the participants.

## Author Contributions

RD and CL initiated and supported the project. FL and MB performed the experiments. FL and RD wrote the manuscript. All authors analyzed the data and commented on the manuscript.

## Conflict of Interest

The authors declare that the research was conducted in the absence of any commercial or financial relationships that could be construed as a potential conflict of interest.

## Footnotes

1 http://www.geneontology.org

2 http://www.genome.jp/kegg

3 http://www.cuilab.cn/hmdd
